# Renin-angiotensin-aldosterone system blockers after KDIGO stage 3 acute kidney injury: use and impact on 2-year mortality in the AKIKI trial

**DOI:** 10.1186/s13054-019-2447-0

**Published:** 2019-04-29

**Authors:** Mathilde Scarton, Anne Oppenheimer, Khalil Chaïbi, Didier Dreyfuss, Stéphane Gaudry

**Affiliations:** 1 AP-HP, Service de Réanimation Médico-Chirurgicale, Hôpital Louis Mourier, F-92700 Colombes, France; 2 Service de Médecine de la Reproduction et Préservation de la fertilité, Hôpital Universitaire Antoine Béclère, Clamart, France; 3 EA 7285 Research Unit ‘Risk and Safety in Clinical Medicine for Women and Perinatal Health’, Versailles-Saint-Quentin University (UVSQ), Versailles, France; 4 AP-HP, Service de Réanimation Médico-Chirurgicale, Hôpital Avicenne, 93000 Bobigny, France; 5 French National Institute of Health and Medical Research (INSERM), UMR_S1155, Remodeling and Repair of Renal Tissue, Hôpital Tenon, F-75020 Paris, France; 6 Present Address: Intensive Care Unit, Hôpital Avicenne, 125 Rue de Stalingrad, 93000 Bobigny, France

Dear Editor,

Acute kidney injury (AKI) carries high mortality and morbidity [1, 2]. Two studies recently suggested the potential benefit of renin-angiotensin system (RAS) blockers (angiotensin-converting enzyme inhibitors (ACEIs) and angiotensin receptor blockers (ARBs)) after AKI [3, 4]. The first one reported a lower mortality after 1 year of follow-up in patients receiving an ACEi or ARB after an episode of AKI (KDIGO stages 1 to 3) at ICU discharge (20/109 (18%) vs 153/502 (31%), *p* = 0.001) [3]. The second one was a retrospective cohort study including adults after an episode of AKI during hospital stay (with 18% only of ICU-patients and only 7% of KDIGO stage 3) [4]. It concluded that exposure to an RAS blocker within the first 6 months after hospital discharge was associated with a 15% decrease in all-cause mortality (HR, 0.85; 95%CI, 0.81–0.89).

We performed an ancillary of the AKIKI trial [5], which included 619 ICU patients with severe AKI (KDIGO stage 3) in order to evaluate the potential effect of RAS blockers on long-term mortality.

All patients discharged alive from ICU were included, and their long-term prognosis (2-year all-cause mortality) was assessed according to treatment with ACEi/ARB at ICU discharge using both univariate and multivariate analyses.

Among 348 patients discharged alive from ICU, 45 (12.9%) received an ACEi/ARB at ICU discharge (see Table 1 for patient characteristics). Patients without ACEi/ARB were more severe as attested by a higher SAPS 3 (*p* = 0.02) and a higher rate of catecholamine infusion (*p* = 0.008) during AKI. However, 2-year all-cause mortality did not significantly differ between the two groups (12/45 (27%) with ACEi/ARB vs 55/303 (18%) without, *p* = 0.18). Mortality risk was not associated with non-prescription of ACEi/ARB after adjustment for prognostic variables (*p* = 0.21) (Table 2).

**Table 1:** Patient characteristics

	ACEi/ARB + (*N* = 45)	ACEi/ARB - (*N* = 303)	*p*
Characteristic at ICU admission			
Age—years	64.4 ± 15.1	63.1 ± 14.7	0.6
Male sex—no. (%)	32 (71)	193 (63)	0.3
Weight—kg	85.8 ± 23.7	82.4 ± 20.5	0.3
Main reason for ICU admission—no. (%)			
Medical	37 (82)	263 (87)	
Surgical, emergency	5 (11)	66 (22)	
Surgical, scheduled	3 (7)	19 (6)	
Serum creatinine before ICU admission	92.0 ± 24.7	83.4 ± 24.0	0.03
Coexisting condition—no. (%)			
Chronic renal failure	6 (13)	26 (9)	0.3
Hypertension	28 (62)	152 (50)	0.1
Diabetes mellitus	3 (7)	31 (10)	0.67
Congestive heart failure	6 (13)	17 (6)	0.05
Ischemic heart disease	8 (18)	26 (9)	0.05
SAPS III at ICU admission	63.0 ± 14.0	68.3 ± 14.3	0.02
Septic shock—no. (%)	26 (58)	128 (42)	0.05
Biological characteristics			
Serum creatinine—µmol/L	203.9 (133.1)	219.6 (136.9)	0.47
Blood urea nitrogen—mmol/L	13.0 (8.4)	14.4 (9.3)	0.34
Serum potassium—mmol/L	4.1 (0.8)	4.3 (0.9)	0.37
Serum bicarbonate—mmol/L	19.9 (5.0)	18.7 (5.7)	0.17
Characteristic of ICU stay			
Physiological support during ICU stay—no. (%)			
Invasive mechanical ventilation	41 (91)	260 (86)	0.33
Vasopressor support (epinephrine or norepinephrine)	31 (69)	257 (85)	0.008
Number of patients who received RRT—no. (%)	28 (62)	211 (70)	0.32
Ventilator duration—median (IQR)	5 (5–13)	5 (4–10)	0.40
Vasopressor-free days—median (IQR)	3 (2–5)	4 (2–7)	0.11
Length of ICU stay—median (IQR)	18 (11–20)	15 (8–22)	0.35
RRT dependence—no. (%)			
At day 28	4 (9)	27 (9)	0.99
At day 60	1 (2)	9 (3)	0.77
Mortality—no. (%[95% CI])			
At day 60	6 (13)	39 (12)	0.73
Creatinine at ICU discharge	183.6 ± 136.4	177.9 ± 150.1	0.81

*ACEi* angiotensin-converting enzyme inhibitors, *ARB* angiotensin receptor blockers, *ICU* intensive care unit, *IQR* interquartile range, *RRT* renal replacement therapy, *CI* confidence interval

**Table 2:** Multivariate analysis

Variable	OR	95%CI	*p*
Age	1.02	[1.00–1.05]	0.04
MacCabe	3.10	[1.64–5.87]	< 0.001
SAPS3	1.04	[1.02–1.07]	< 0.01
CKD	1.99	[0.77–4.89]	0.14
Congestive heart failure	2.12	[0.66–6.43]	0.19
History of acute stroke	1.91	[0.80–4.38]	0.13
ACEi/ARB at ICU discharge	1.71	[0.71–3.90]	0.21

*CKD* chronic kidney disease

We acknowledge that our study did not assess introduction nor interruption of ACEi/ARB after ICU discharge. One consequence of the severity of AKI in our study is that most patients had not fully recovered at ICU discharge. In this condition, physicians in charge could be reluctant to initiate ACEi/ARB in ICU but treated the patients later.

Our study does not confirm findings from two recent studies [3, 4]. This discrepancy could be explained by a different population (less severe AKI in previous studies) and/or a lack of power of our study but in any case warrant the performance of a randomized controlled trial of ACEi/ARB at ICU discharge after an episode of severe AKI.

## Abbreviations

ACEIs: Angiotensin-converting enzyme inhibitors
AKI: Acute kidney injury
ARBs: Angiotensin receptor blockers
ICU: Intensive care unit
KDIGO: Kidney Disease Improving Global Outcomes
RAS: Renin-angiotensin system
